# Validation of a Swahili version of the World Health Organization 5-item well-being index among adults living with HIV and epilepsy in rural coastal Kenya

**DOI:** 10.1186/s41256-018-0081-z

**Published:** 2018-09-10

**Authors:** Esther Chongwo, Derrick Ssewanyana, Carophine Nasambu, Patrick N. Mwangala, Paul M. Mwangi, Moses K. Nyongesa, Charles R. Newton, Amina Abubakar

**Affiliations:** 1 Centre for Geographic Medicine Research Coast, Kenya Medical Research Institute (KEMRI), P.O Box 230, Kilifi, Kenya; 2 Utrecht Centre for Child and Adolescent Studies, Utrecht University, P.O Box 80140, 3508 TC Utrecht, The Netherlands; 3 Department of Public Health, Pwani University, P.O Box 195, Kilifi, Kenya; 4 Department of Psychiatry, Warneford Hospital, University of Oxford, Oxford, OX3 7JX UK

**Keywords:** Wellbeing, Validation, HIV, Epilepsy, Psychometrics, WHO-5 item index

## Abstract

**Objective:**

The purpose of this study was to evaluate the psychometric properties of the World Health Organization’s five item well-being index (WHO-5) when administered to adults living with HIV or epilepsy in a rural setting at the coast of Kenya.

**Methods:**

A case control study design was conducted among 230 adults aged 18–50 years, who comprised 147 cases (63 living with epilepsy and 84 living with HIV) and 83 healthy controls. The participants were administered to a face-to-face interview during which they completed the Swahili version of WHO-5 well-being index, the Major Depression Inventory (MDI) and responded to some items on their socio-demographic characteristics. Analysis to assess internal consistency, construct validity, discriminant validity, and convergent validity of the Swahili version of WHO-5 well-being index was conducted. A multivariate regression was carried out to assess the association between psychological wellbeing (assessed using Swahili version of WHO-5 well-being index) and having a chronic illness (HIV or epilepsy).

**Results:**

The Swahili version of WHO-5 well-being index demonstrated good internal consistency with Cronbach alpha ranges of 0.86–0.88 among the three study groups. The tool had good discriminant validity. A one factor structure of the tool was obtained from confirmatory factor analysis (overall Comparative Fit Index = 1.00, Tuckler Lewis Index = 1.01, Root Mean Square of Error Approximation = 0.00). Living with HIV or epilepsy in comparison to being a healthy control was significantly associated with greater odds of having sub-optimal psychological wellbeing.

**Conclusion:**

Our findings demonstrate that the Swahili version of WHO-5 well-being index has good psychometric properties and is appropriate for use to evaluate psychological well-being among adults living with chronic conditions such as HIV or epilepsy from a rural low resource setting in Kenya. Given its brevity and ease of use, the Swahili version of WHO-5 well-being index could potentially be used by lay workers and other paraprofessional to monitor psychological well-being among chronically ill adults in resource poor settings.

## Introduction

Chronic illnesses such as HIV or epilepsy are often accompanied by psychological problems which subsequently contribute to a low quality of life [1, 2]. Identification of these problems through screening using psychological tools in this population group would be integral in optimizing their care. In low resource settings such as Kenya, psychological assessments are rarely performed partly due to insufficient resources and lack of validated psychological measures [3]. Recent efforts towards development of brief, simple, cheap and easy to administer tools have been made by the World Health Organization (WHO).

The 5-item WHO well-being index (WHO-5) [4] measures psychological well-being and was developed from the 10-item WHO well-being index (WHO-10), which was originally a 28-item scale [5]. The WHO-5 was first published in 1998 after WHO meeting in Stockholm where it was presented as part of a project on evaluation of well-being in primary health care unit patients. Since then, the original version of this tool has been translated into more than 30 languages [6]. The tool has been demonstrated to have adequate validity for measurement of well-being in a wide range of patient groups such as people living with diabetes, breast cancer, cardiac disease, and neurological conditions as well as among paediatric and geriatric sub-populations [6].

In various validation studies, the WHO-5 well-being index has demonstrated good psychometric properties with high internal consistency and validity [6–8]. Confirmatory factor analysis of the tool has demonstrated that the sub-items measure a unidirectional factor structure [9].However, all these studies originate from high income countries and there have not been any evaluating its psychometric properties in East and Central Africa, where Swahili is the lingua franca spoken by up to 150 million people.

The objective of this study was to examine the reliability and validity (construct, divergent and discriminatory) of the Swahili version of WHO-5 well-being index in screening psychological well-being in the epilepsy and HIV subpopulations. We also aimed at assessing the association between psychological wellbeing and disease status (i.e. living with epilepsy and HIV).

## Methods

### Study site and population

The study was conducted at the Centre for Geographic Medical Research, (CGMRC) located at the Kenyan coast in Kilifi. The Centre is located within Kilifi County Hospital (KCH) and has facilities for performing neuropsychological studies. Within the hospital, there is a clinic offering specialized care to people living with epilepsy and a Comprehensive Care and Research Centre (CCRC) offering care for people living with HIV. from where recruitment of the study participants took place. Established in 2000, the Kilifi Health and Demographic Surveillance System (KHDSS) which has 260,000 residence conducts census 4-monthly allowing for adequate follow-up of study subjects [10] was used to recruit health controls from the community.

The native language of the people residing in the area is Mijikenda language (commonly Kigiriama) and the Kiswahili national language. It is estimated that 58% of the residents of Kilifi County live below the poverty line and a third (36%) have not attained any formal education [11]. Epilepsy (prevalence 4.5 per 1000) and HIV (prevalence 44 per 1000) are common in this region [12–14].

### Participants

Stratified random sampling was employed to recruit the study subjects. The study sample size was 230 which is in agreement with the guidelines for standard psychometric analysis whereby the least sample size should be 100 estimates [15]. The adults with confirmed diagnosis of HIV were recruited from Kilifi County Hospital’s CCRC, the adults with epilepsy from the epilepsy clinic and the community controls were recruited from the KHDSS. Using existing patient records from the epilepsy clinic, a random sample of 100 patients was identified. From the CCRC, an attending clinician identified eligible participants whenever they visited the clinic for care. The identified patients from both clinics were either consented at the clinic or followed up to their households to seek their consent for participation in the study. A random sample of 100 adult controls were also identified through the KHDSS database and latter approached at their homes to seek their consent for participation. The general inclusion criteria of the participants was: a person aged 18–50 years; education level not more than primary level; fluent in Swahili language; and provision of informed consent. Cases were included if they had a confirmed diagnosis of epilepsy or HIV. For the control group, they must not have had a history of chronic illnesses. Participants who were too sick to take part in the study were excluded.

### Procedure

Upon consenting, the study participants were referred to the neuro-assessment unit at the CGMRC whereby the examination was carried out. Administration of the neuropsychological measures was done by trained research staff. All the study participants completed a socio-demographic questionnaire, the Swahili version of WHO-5 well-being index and the Major Depression Inventory (MDI).

### Measures

#### The WHO-5 well-being index

This is a self-administered psychological well-being measure although in our study it was administered as an oral interview. It comprises of five items of: i) ‘I have felt cheerful and in good spirits’; ii) ‘I have felt calm and relaxed’; iii) ‘I have felt active and vigorous’; iv) ‘I woke up feeling fresh and rested’; and v) ‘My daily life has been filled with things that interest me.’ These items positively assesses the degree of well-being during the past two weeks. They are scored on a 6-point Likert scale whereby ‘0’ stands for (at no time) and ‘5’ for (at all time). The raw total score ranges from 0 to 25 with a higher score being an indication of high state of psychological well-being [4]. The total raw score is multiplied by 4 to give the final score whereby, 0 represents the lowest level of well-being and 100 represents the highest. Studies conducted among participants with various health conditions have shown good diagnostic accuracy (sensitivity of 0.86 and specificity of 0.81) of the WHO-5 well-being index while using a cut-off score of 50 [6]. Scores less than 50 indicate sub-optimal wellbeing and those above 50 indicate optimal wellbeing. This scale was forward translated from its original English language to Swahili by two independent translators. It was then back translated by one independent bilingual translator who was unaware of the original English version. A harmonization/adjudication meeting was then held, including all the translators and the research team members fluent in both languages, to discuss and resolve the differences in the translations and reach a consensus on the best wording.

#### Major depression inventory (MDI)

A self-rated questionnaire developed to measure state of moods in the past two weeks [16]. The 10-item are scored on a 6-point Likert scale. The total score ranges from 0 which indicates no depression to a score of 50 which indicates extreme depression. A total score of 0–20 indicates no or doubtful depression; 21–25 indicates mild depression; 26–30 indicates moderate depression; and 31–50 indicates severe depression. The MDI has been found to have good psychometric characteristics when used among adolescents and young adults in Kilifi [17].

Demographic variables: Items were developed to capture the age, sex, marital status, and occupation, level of education and socio-economic status of the study participants.

### Statistical analyses

Data analyses was done in STATA version 14 and R program version 3.4.1. Assessment for internal consistency was done using Cronbach’s alpha and omega [18]. Correlational analysis between scores from the Swahili version of WHO-5 well-being index and MDI was done to assess divergent validity while discriminant validity was assessed by analysis of variance (ANOVA) followed by a post hoc analysis to asses for variations across study groups. A *p*-value of ≤0.05 was considered as cutoff for statistically significant results. Confirmatory factor analysis (CFA) with diagonally weighted least squares was conducted using structural equation modelling to determine the factor structure of the WHO-5 tool. From CFA, acceptable model fit was obtained if the root mean squared error of approximation (RMSEA) was < 0.06 and if the Tucker–Lewis Index (TLI) and Comparative Fit Index (CFI) were > 0.9 [19, 20]. A binary outcome of wellbeing (Optimal psychological wellbeing (> 50) and Sub-optimal psychological wellbeing (≤50)) was generated. A multivariate logistic regression model was fitted for the association between psychological wellbeing (assessed using Swahili version of WHO-5 well-being index) and having a chronic illness (HIV or epilepsy). This model adjusted for participants’ sex, age, socio-economic status and marital status.

### Ethical considerations

The Kenya Medical Research Institute (KEMRI) Scientific and Ethics Review Unit (SERU) approved this study to be conducted (KEMRI/SERU/CGMRC-C/030/3187). Prior to data collection, written informed consent was obtained from all the study participants. Anonymity and confidentiality was ensured by using participant study numbers as opposed to personal identifiers, and by conducting interviews in a quiet and private environment.

## Results

Of the 230 study participants, 64% were cases (i.e. people living with HIV or epilepsy) and majority (66.5%) were females. The mean age of the participants was 34.8 (SD = 8.8). There were significant differences (F = 1.6, *p* = 0.03) in age with adults living with HIV being the oldest group years (40.1, SD = 6.4), followed by the community controls (33.8, SD = 9.5), and the adults with epilepsy were youngest (28.8, SD = 6.1). A description of the socio-demographic characteristics of the participants is summarized in Table 1 below.

**Table 1:** Socio-demographic characteristics of the study participants

Variable	Controls n (%)	Epilepsy n (%)	HIV n (%)	*p*-value
Gender				
Male	30(36.1)	29(46.0)	18(21.4)	0.006
Female	53(63.9)	34(54.0)	66(78.6)	
Education level				
Primary complete	49(59.0)	8(12.7)	16(19.0)	
Primary incomplete	26(31.3)	40(63.5)	47(56.0)	< 0.001
No education	8(9.6)	15(23.8)	21(25.0)	
Occupation				
Informal	43(51.8)	27(42.9)	65(77.4)	
Formal	5(6.0)	0(0.0)	1(1.2)	< 0.001
Unemployed	35(42.2)	36(57.1)	18(21.4)	
Marital status				
Never married	20(24.1)	53(84.1)	8(9.5)	< 0.001
Married	59(71.1)	7(11.1)	47(56.0)	
Separated	3(3.6)	1(1.6)	5(6.0)	
Divorced	1(1.2)	1(1.6)	6(7.1)	
Widowed	0(0)	1(1.6)	18(21.4)	

The Swahili version of WHO-5 well-being index demonstrated good internal consistency in all the three study groups. The Cronbach alphas were all high and they were: 0.87 (95% CI, 0.82–0.91) among the control group; 0.88 (95% CI, 0.83–0.92) among the adults living with HIV; and 0.86 (95% CI, 0.75–0.91) among the adults living with epilepsy.

**Fig. 1:**
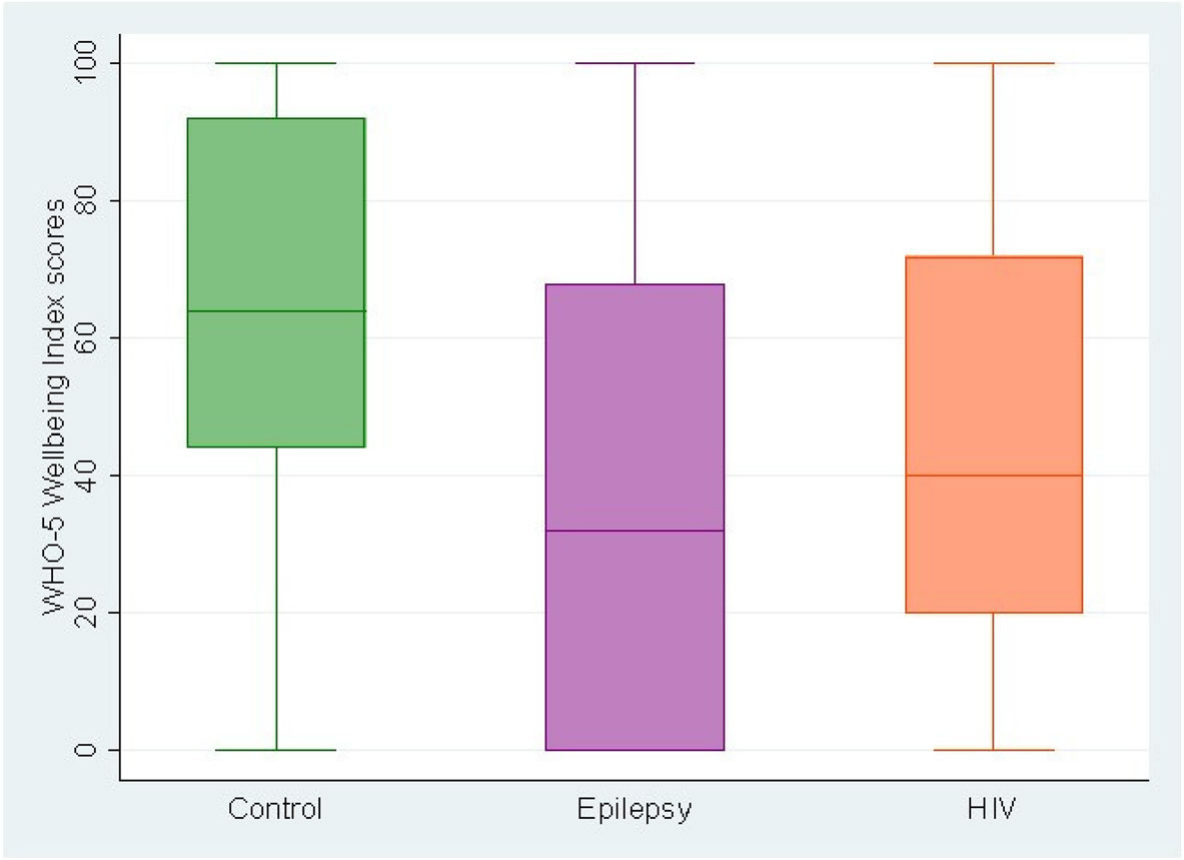
A box plot of WHO-5 mean scores across the three participant study groups

The results from the discriminant validity of the Swahili version of WHO-5 well-being index indicated that this tool showed statistical differences (F = 15.9, *p* value < 0. 001) in performance across the three groups (i.e. people with HIV, epilepsy and controls). Raw mean scores on the Swahili version of WHO-5 well-being index were highest among healthy controls (62.3, SD = 28.8), followed by adults living with HIV (45.6, SD = 28.4), and lastly among adults living with epilepsy (34.7, SD = 32.9) as show in Fig. 1 below. Results from ANOVA post-hoc analysis indicated that the differences in mean scores on the Swahili version of WHO-5 well-being index were significant between; adults living with HIV and those living with epilepsy; adults living with HIV and the healthy community controls; and adults living with epilepsy and the healthy community controls.

Furthermore, a statistically significant negative correlation (− 0.26, *p* < 0.001) the Swahili version of WHO-5 well-being index and MDI confirmed divergent validity of the Swahili version of WHO-5 well-being index.

Results from CFA with diagonally weighted least squares of WHO-5 wellbeing index resulted in a one-factor structure that was adequately fitting across all the study participants’ groups and the results are summarized in Table 2.

**Table 2:** Fit indices from confirmatory factor analysis of the WHO-5 well-being index summarized by chronic illness status

Study groups	Chi-square	RMSEA	RSMR	CFI	TLI
Controls	0.89	0.00	0.03	1.00	1.00
Epilepsy	0.98	0.00	0.03	1.00	1.06
HIV	0.03	0.14	0.04	0.97	0.94
Overall	0.81	0.00	0.04	1.00	1.01

We further conducted a multivariate logistic regression analysis to investigate the association between psychosocial wellbeing (using the Swahili version of WHO-5 well-being index, Optimal psychological wellbeing (> 50) and Sub-optimal psychological wellbeing (≤50)) and chronic illness status (i.e. epilepsy, HIV and health group) of the participants. Having a chronic condition (epilepsy or HIV) as compared to being a healthy community control was significantly (*p* = 0.004) associated with greater odds of having sub-optimal psychological wellbeing, while adjusting for participants’ sex, age, socio-economic status and marital status. The odds of having sub-optimal psychological wellbeing were highest among adults living with epilepsy. The results from the multivariate analysis are summarized in Table 3 below.

**Table 3:** Results from a multivariate logistic regression of psychological wellbeing and chronic illness status of the study participants

Variable	Odd Ratio (95% CI)	*p*-value
Chronic Illness		0.004
Controls	1	
Adults living with Epilepsy	4.04 (1.68, 9.69)	0.002
Adults living with HIV	2.07 (1.02, 4.20)	0.044
Sex		
Females	1	
Males	1.08 (0.5, 1.99)	0.798
Age (years)	1.04 (1.00, 1.08)	0.037
Social economic status (assets index)	0.92 (0.75, 1.34)	0.445
Marital status		0.757
Never married	1	
Married	0.69 (0.28, 1.48)	0.304
Separated	1.00 (0.21, 4.65)	0.997
Divorced	0.94 (0.18, 4.86)	0.940
Widowed	1.09 (0.29, 4.05)	0.900

*CI* Confidence Interval, *%* Percentage

## Discussion

The purpose of this study was to examine the psychometric properties of the Swahili version of WHO-5 well-being index [4] in adults living with HIV or epilepsy from a rural setting in coastal Kenya.

Our findings demonstrate that the Swahili version of the WHO-5 well-being index has good psychometric properties. This tool demonstrated good internal consistency ranging between 0.86–0.88 across the 3 study groups in our study. These findings concur with what has been reported in other studies for instance in Spain where a Spanish version of the WHO-5 well-being index showed good internal consistency of 0.86 when used in an elderly population [8]. An alpha of 0.82 was obtained when the Dutch version of the tool was used among an adolescent sub-population living with type 1 diabetes in Netherlands [9].

Furthermore, we found a unidimensional structure of the Swahili version of the WHO-5 well-being index. This unidimensional structure was maintained across the three study groups despite having significant differences in socio-demographic characteristics of sex, educational level, occupation and marital status. This demonstrates that the WHO-5 well-being index is a flexible tool for use in groups with varying chronic health conditions and socio-demographic characteristics. This finding confirms that indeed a single underlying factor (which in this case is psychological wellbeing) is assessed by the WHO-5 well-being index among our study population.

Our results of a negative correlation between the scores on the Swahili version of the WHO-5 well-being index and the scores on the MDI show that good psychosocial wellbeing is negatively associated with depression, and this finding is plausible [21]. Therefore, these results demonstrate good divergent validity of the Swahili version of the WHO-5 well-being index. Similar findings of a negative correlation between the WHO-5 well-being index and a measure of depression (Center for Epidemiologic Studies Depression Scale (CES-D)) were reported in another study among adolescents with type 1 diabetes [9].

Our findings also show that psychosocial wellbeing (measured using the Swahili version of the WHO-5 well-being index) is better among the adults living with HIV compared to those living with epilepsy in Kilifi. The reasons for this difference are beyond the scope of our study, however, this may be partly due to the large epilepsy treatment gap in Kilifi (estimated at 62% [13]). Furthermore, we found that the healthy controls had better scores on the Swahili version of the WHO-5 well-being index compared to the two groups with chronic illness, and these findings are coherent with those from other studies that have compared participants with chronic illnesses and healthy controls [22, 23]. This result also further demonstrates that the Swahili version of the WHO-5 well-being index has good discriminant validity.

Our study had some limitations. Firstly, this data was obtained from self-reported measures which may sometimes result into social desirability bias and/or recall bias. Secondly, we did not assess the test-retest reliability of the Swahili version of the WHO-5 well-being index. However, the major strength of the study is the fact that we involved three study groups and utilized a variety of measures besides the tool of interest (Swahili version of the WHO-5 well-being index) which thus improved the fidelity and reliability of our findings. Secondly, this is the only study to assess the psychometric properties of the WHO-5 well-being index among a chronically ill adult sub-population in the rural Kenyan setting.

Future studies are needed to explore the test-retest and inter-rater reliability of the WHO-5 well-being index when used by lay health workers within low resource settings.

## Conclusions

The Swahili version of the WHO-5 well-being index demonstrates good psychometric properties such as internal consistency; construct, discriminatory and divergent validity, when used among adults living with HIV and epilepsy in a rural setting in coastal Kenya. This further accentuates the potential of its usefulness especially for healthcare workers in low resource settings in assessing psychosocial wellbeing of chronically ill patients during routine care and management. It’s briefness and yet good psychometric quality makes it a feasible tool to incorporate into existing healthcare data tools especially for use by lay health workers.

## Abbreviations

ANOVA: Analysis of variance
CCRC: Comprehensive Care and Research Centre
CFA: Confirmatory factor analysis
CFI: Comparative Fit Index
CGMRC: Centre for Geographic Medical Research
HIV: Human Immuno-deficiency Virus
KEMRI: Kenya Medical Research Institute
KHDSS: Kilifi Health and Demographic Surveillance System
MDI: Major Depression Inventory
SD: Standard Deviation
TLI: Tucker–Lewis Index
WHO: World Health Organization
